# Infection-Associated Transient Neutropenia Mimicking Methimazole-Induced Agranulocytosis in Graves' Disease: A Diagnostic Dilemma

**DOI:** 10.7759/cureus.103363

**Published:** 2026-02-10

**Authors:** Saraswathi Saiprasad, Narayana Swamy, Theresa Cao, Sriharika Gottipolu

**Affiliations:** 1 Diabetes and Endocrinology, Baylor Scott & White Health, Fort Worth, USA; 2 Rheumatology, Baylor Scott & White Health, Fort Worth, USA; 3 Internal Medicine, Baylor Scott & White Health, Fort Worth, USA

**Keywords:** agranulocytosis, antithyroid drugs, graves’ disease, methimazole, neutropenia, postpartum

## Abstract

Methimazole is the preferred antithyroid drug for Graves’ disease but is rarely associated with agranulocytosis, a potentially life-threatening adverse effect that traditionally mandates permanent drug discontinuation. Agranulocytosis is defined by an absolute neutrophil count (ANC) below 500 cells/µL. We report a case of severe neutropenia occurring during methimazole therapy in a young woman with Graves’ disease that closely mimicked methimazole-induced agranulocytosis but was ultimately consistent with infection-associated transient neutropenia. While receiving methimazole, the patient developed acute tonsillitis with a nadir ANC of 0.06 × 10³/µL, prompting immediate discontinuation of therapy. Rapid hematologic improvement was observed within approximately 24 hours, with continued recovery of neutrophil counts as the infection resolved. In contrast, thyroid indices worsened following methimazole withdrawal, necessitating a time-sensitive management decision. After objective evidence of hematologic recovery and careful reassessment of causality, methimazole was cautiously reintroduced at a reduced dose under predefined stopping rules and close laboratory monitoring, resulting in sustained neutrophil normalization and continued thyroid control. Reintroduction of antithyroid drug therapy is not standard practice in confirmed antithyroid drug-induced agranulocytosis, and both methimazole and propylthiouracil remain contraindicated in that setting. This case does not alter established recommendations but highlights practical clinical features, particularly ANC trajectory, timing of recovery, and infectious context, that may help distinguish classic drug-induced agranulocytosis from transient infection-related neutropenia and support highly individualized, patient-centered decision-making when ideal care pathways are constrained.

## Introduction

Graves’ disease is the most common cause of hyperthyroidism in young adults and is typically managed with antithyroid drugs, radioactive iodine ablation, or thyroidectomy, depending on disease severity, patient characteristics, and regional practice patterns [1,2]. Antithyroid drugs remain first-line therapy in many clinical settings because of their effectiveness, reversibility, and avoidance of definitive thyroid destruction. Among available agents, methimazole is preferred over propylthiouracil because of its longer duration of action, superior efficacy, and lower risk of severe hepatotoxicity [2,3]. Clinical practice guidelines from both the American Thyroid Association and the European Thyroid Association recommend methimazole as the initial antithyroid agent for most patients with Graves’ disease [1,3].

Although methimazole is generally well tolerated, rare but potentially life-threatening adverse effects can occur. Agranulocytosis is the most feared complication of antithyroid drug therapy and is defined as an absolute neutrophil count (ANC) below 500 cells/µL. In large observational series, agranulocytosis has been reported in approximately 0.35%-0.37% of patients treated with methimazole or propylthiouracil [2]. This complication most commonly occurs within the first 90 days of therapy, although delayed presentations have also been described [2,4]. Because recurrence after re-exposure has been reported, current guidelines recommend immediate and permanent discontinuation of methimazole once true antithyroid drug-induced agranulocytosis is confirmed, with avoidance of propylthiouracil due to potential cross-reactivity [1,2].

In contrast, infection-associated transient neutropenia is a well-recognized phenomenon and may closely mimic antithyroid drug-induced agranulocytosis both clinically and biochemically [5]. Acute infectious or inflammatory states can result in profound but reversible neutropenia through immune-mediated peripheral destruction or transient bone marrow suppression. In such cases, neutrophil counts may fall to levels meeting laboratory criteria for agranulocytosis yet recover rapidly as the underlying illness resolves. Consequently, the temporal trajectory of neutrophil recovery, rather than the absolute nadir alone, often provides the most useful clue to etiology.

Adding to this complexity, Graves’ disease itself has been associated with mild baseline leukopenia or neutropenia in some patients even prior to initiation of antithyroid drug therapy. This underlying hematologic variability may further complicate the interpretation of leukopenia detected during treatment and increase the risk of misattributing transient or infection-related neutropenia to drug toxicity.

Distinguishing true antithyroid drug-induced agranulocytosis from transient infection-associated neutropenia is therefore clinically critical, yet in practice may not always be immediately possible at the time of presentation. Current standards of care appropriately recommend permanent discontinuation of methimazole once true agranulocytosis is confirmed, and this case does not challenge that principle. Rather, in this patient, the rapid recovery of neutrophil counts and clear temporal association with acute infection supported an alternative etiology, permitting an exceptionally cautious and closely monitored reintroduction of methimazole as an individualized decision. Premature and permanent discontinuation of methimazole may expose patients to uncontrolled thyrotoxicosis or necessitate definitive therapies that may not be immediately feasible or desirable. This diagnostic challenge is further amplified in real-world settings where access to frequent laboratory monitoring, hospitalization, or definitive therapy is limited by financial or logistical constraints.

We report a case of severe neutropenia occurring during methimazole therapy for Graves’ disease that closely mimicked antithyroid drug-induced agranulocytosis. Rapid hematologic recovery, a clear temporal association with an acute infectious illness, and sustained neutrophil normalization under cautious dose adjustment supported a diagnosis of infection-associated transient neutropenia rather than classic immune-mediated drug toxicity. This case highlights practical clinical features that aid in differentiation while reinforcing adherence to established guidelines and emphasizing the importance of disciplined, patient-centered clinical judgment in complex real-world scenarios.

## Case presentation

A woman in her mid-20s presented for endocrine evaluation approximately one year postpartum with progressive symptoms of hyperthyroidism, including heat intolerance, diaphoresis, tremor, palpitations, diarrhea, anxiety, and significant unintentional weight loss. She had no prior history of thyroid disease. Thyroid function tests had been obtained at multiple laboratories over time; reference ranges are reported as provided by the performing laboratory and were interpreted in the appropriate clinical context. Six months prior to presentation, thyroid testing demonstrated a thyroid-stimulating hormone (TSH) level of 0.262 µIU/mL (reference range 0.46-4.46 µIU/mL) with a free thyroxine (FT4) level of 0.95 ng/dL (reference range 0.70-1.48 ng/dL), consistent with subclinical hyperthyroidism while the patient was not receiving antithyroid therapy.

At presentation, laboratory evaluation demonstrated overt biochemical hyperthyroidism, with a TSH level < 0.005 µIU/mL (reference range 0.27-4.20 µIU/mL) and an FT4 level of 2.86 ng/dL (reference range 0.92-1.68 ng/dL). Autoimmune evaluation confirmed Graves’ disease, with elevated thyroid-stimulating immunoglobulin (TSI) at 221% (reference <140% baseline) and thyrotropin receptor antibodies (TRAb) at 2.03 IU/L (reference <2.0 IU/L). Thyroid peroxidase antibody was markedly elevated at 569 IU/mL (reference <9 IU/mL), consistent with coexisting autoimmune thyroiditis; however, the clinical and biochemical phenotype was dominated by hyperthyroidism due to Graves’ disease.

**Figure 1:**
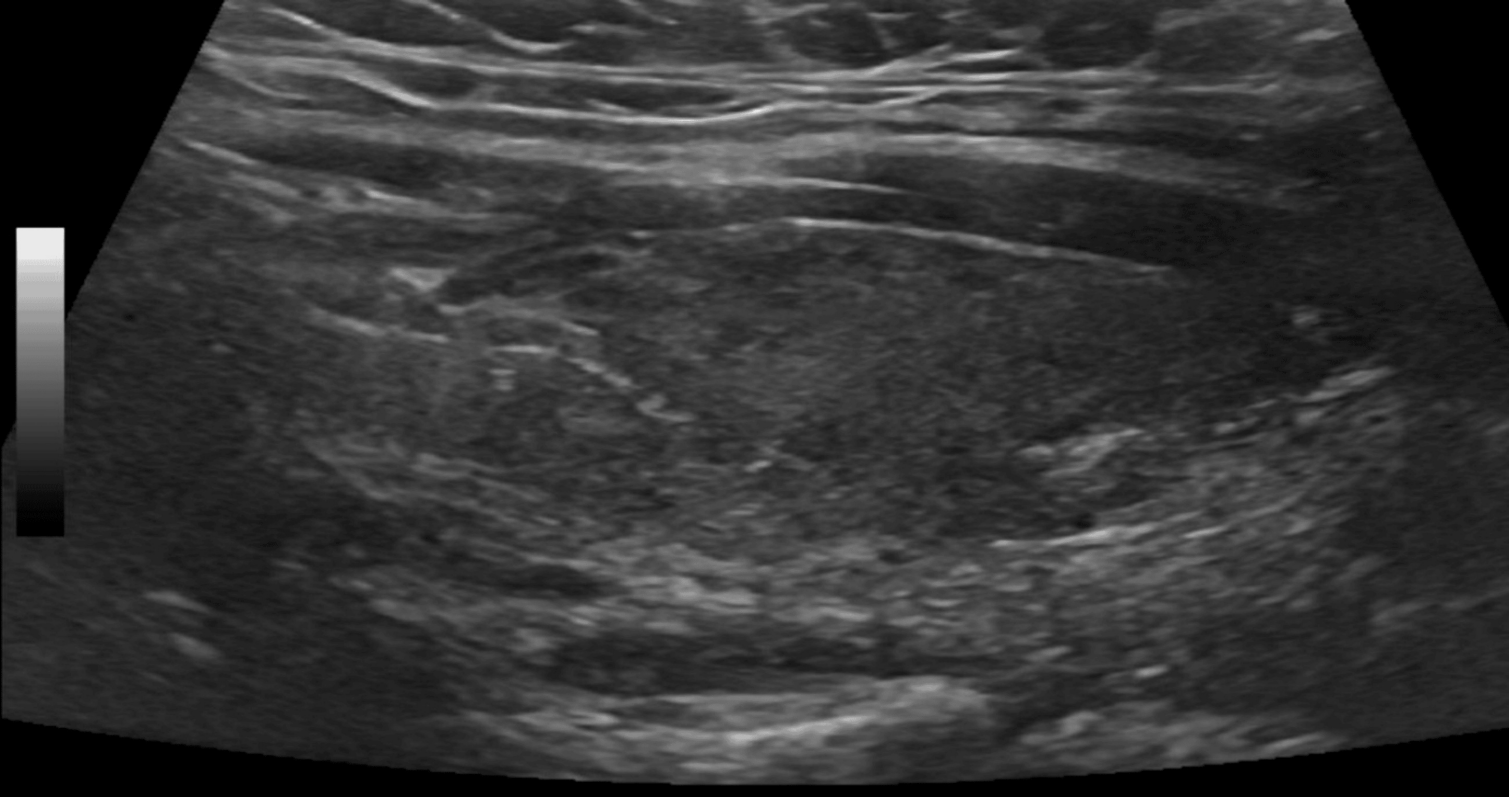
Longitudinal ultrasound image of the right thyroid lobe showing homogeneous echotexture without nodules

**Figure 2:**
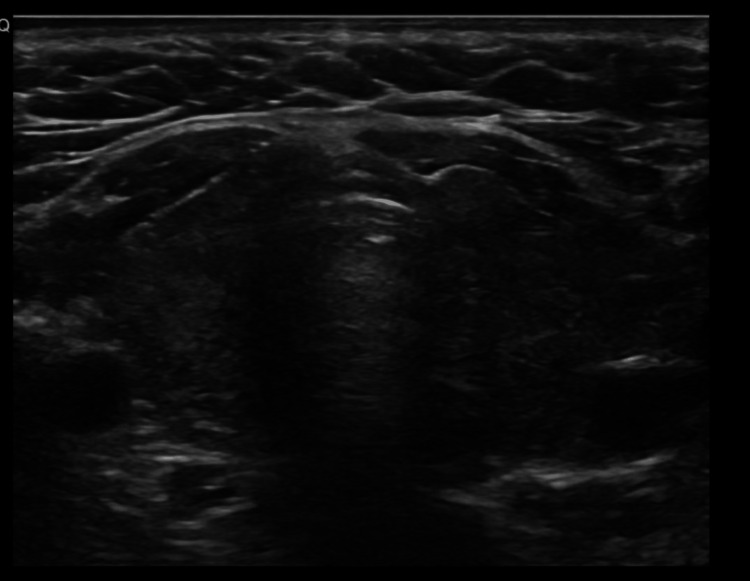
Transverse thyroid ultrasound image showing homogeneous echotexture of both thyroid lobes without nodules

On physical examination, the patient was tachycardic but afebrile. There was no evidence of thyroid-associated ophthalmopathy. The thyroid gland was not significantly enlarged, with no palpable nodules or audible thyroid bruit. There were no signs of pretibial myxedema or acropachy. Thyroid ultrasonography demonstrated a homogeneous gland without nodules (Figures 1, 2).

Methimazole was initiated at a dose of 15 mg daily (total weekly dose 105 mg), along with a beta-adrenergic blocker for symptomatic control. Approximately one month later, thyroid indices demonstrated clear biochemical improvement, with a TSH of 0.03 µIU/mL and an FT4 of 0.94 ng/dL, confirming an appropriate therapeutic response, and the beta-blocker was subsequently discontinued.

Approximately six weeks after methimazole initiation, within the recognized high-risk window for antithyroid drug-associated agranulocytosis, the patient presented to the emergency department with acute tonsillitis and odynophagia. Baseline hematologic indices obtained six months earlier had been within normal limits, with a white blood cell (WBC) count of 4.4 × 10³/µL and an ANC of 1.5 × 10³/µL. At the time of emergency department presentation, laboratory evaluation revealed marked leukopenia and profound neutropenia, with a WBC count of 1.5 × 10³/µL and an ANC of 0.06 × 10³/µL, meeting laboratory criteria for agranulocytosis. Methimazole was immediately discontinued.

**Figure 3:**
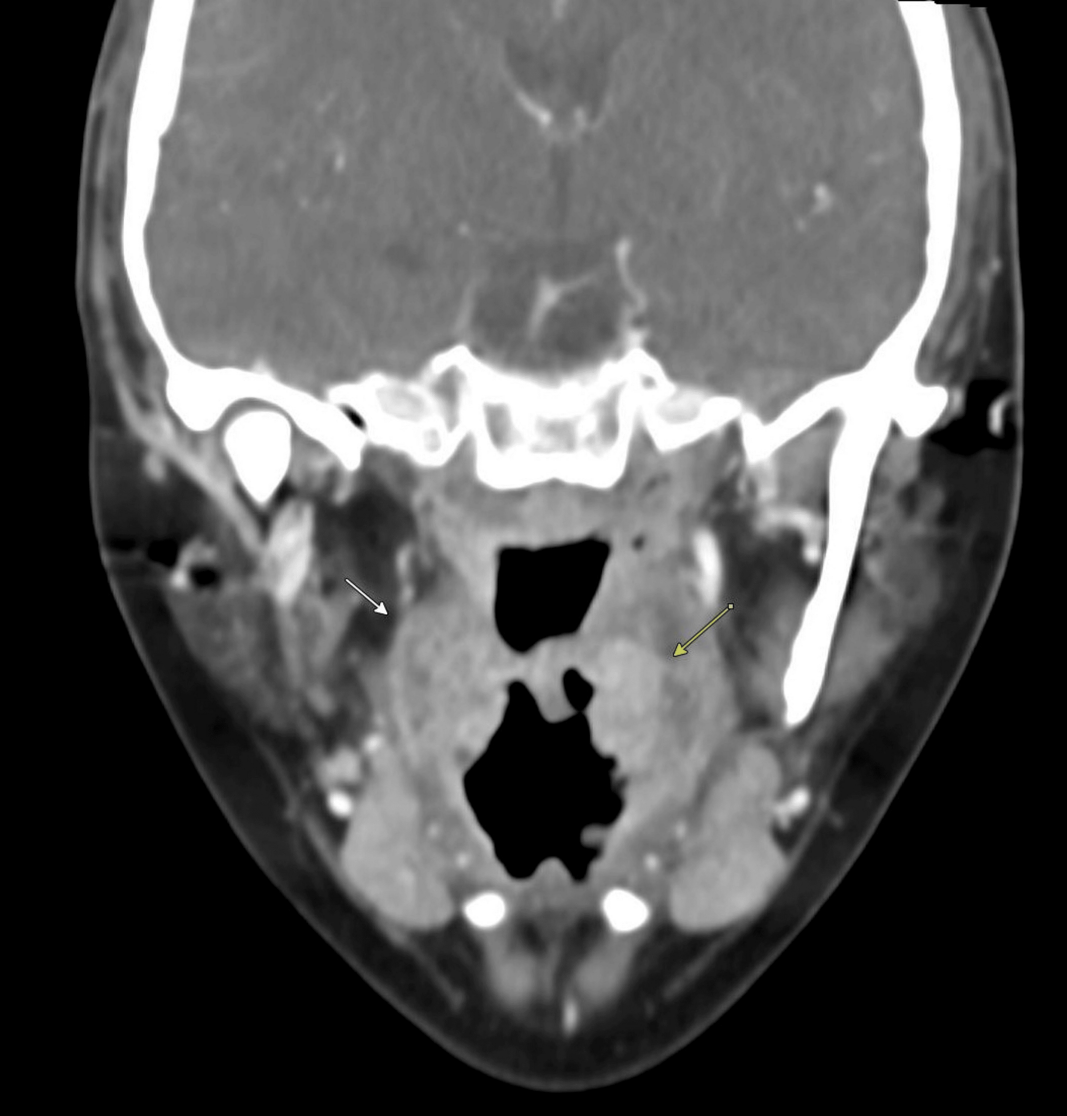
Coronal computed tomography image demonstrating bilateral palatine tonsillar enlargement consistent with acute tonsillitis (arrows)

**Figure 4:**
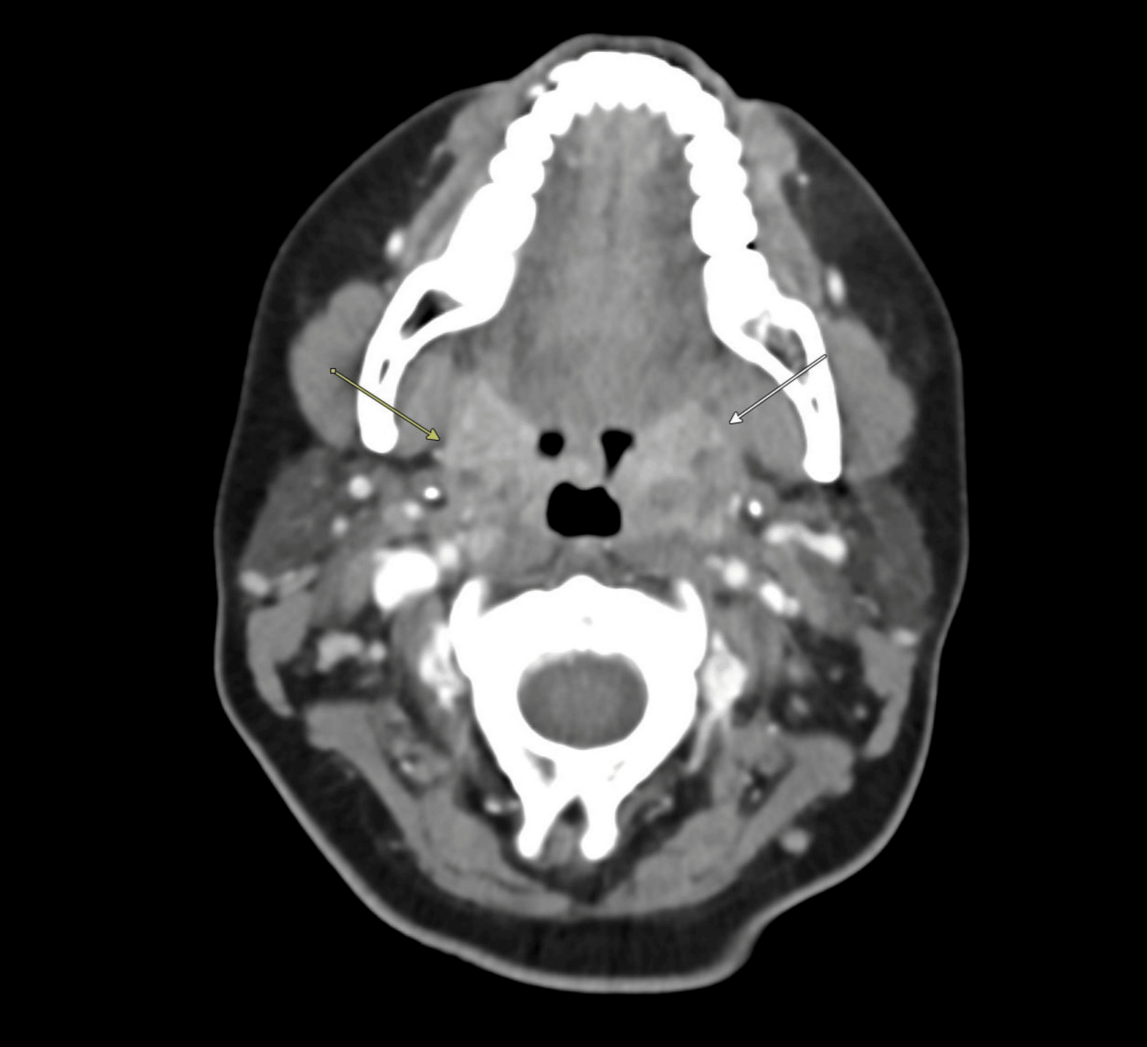
Transverse computed tomography image of the neck demonstrating bilateral palatine tonsillar enlargement consistent with acute tonsillitis (arrows)

**Figure 5:**
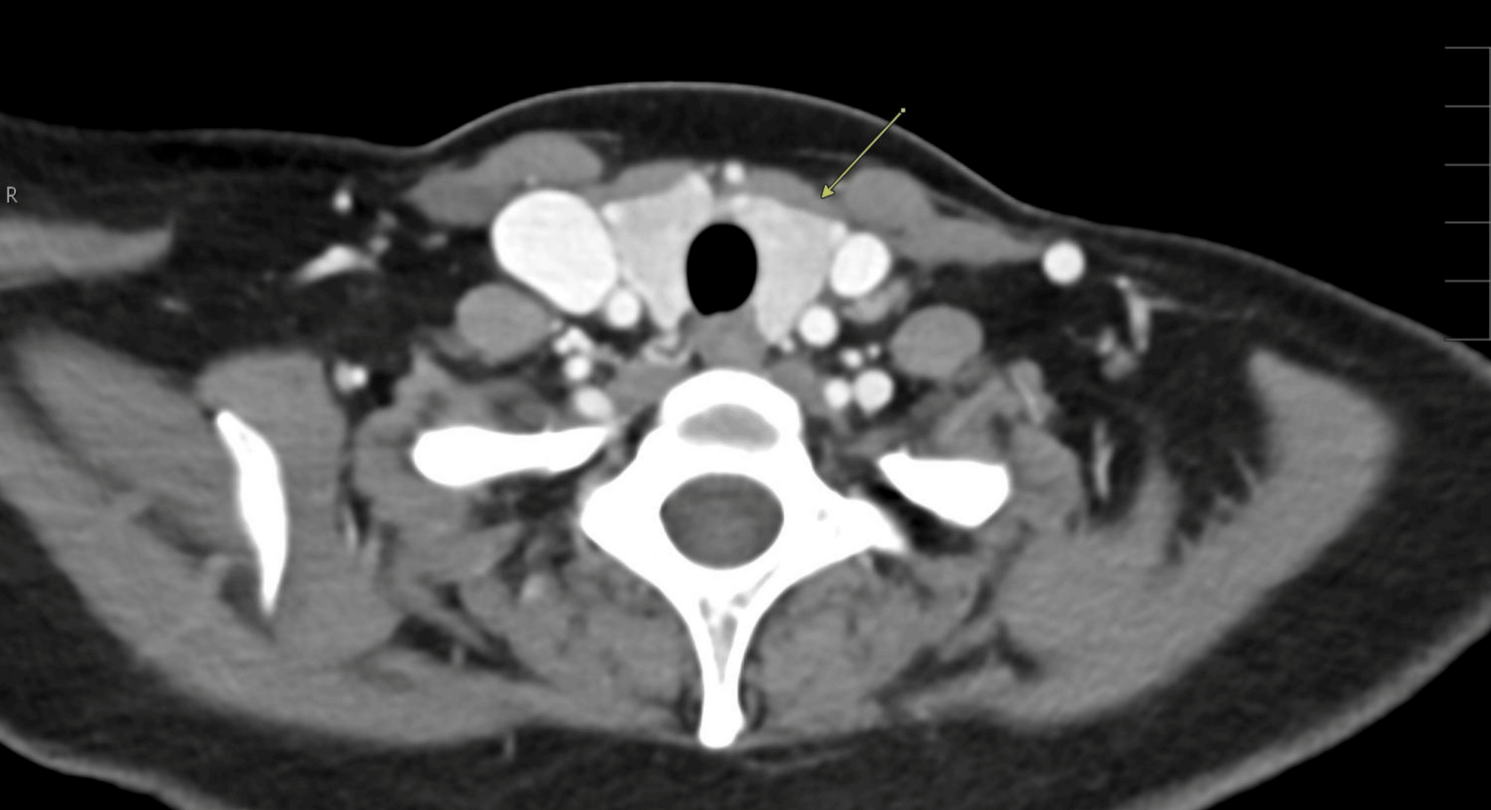
Transverse computed tomography image demonstrating homogeneous appearance of the bilateral thyroid lobes (arrow)

**Figure 6:**
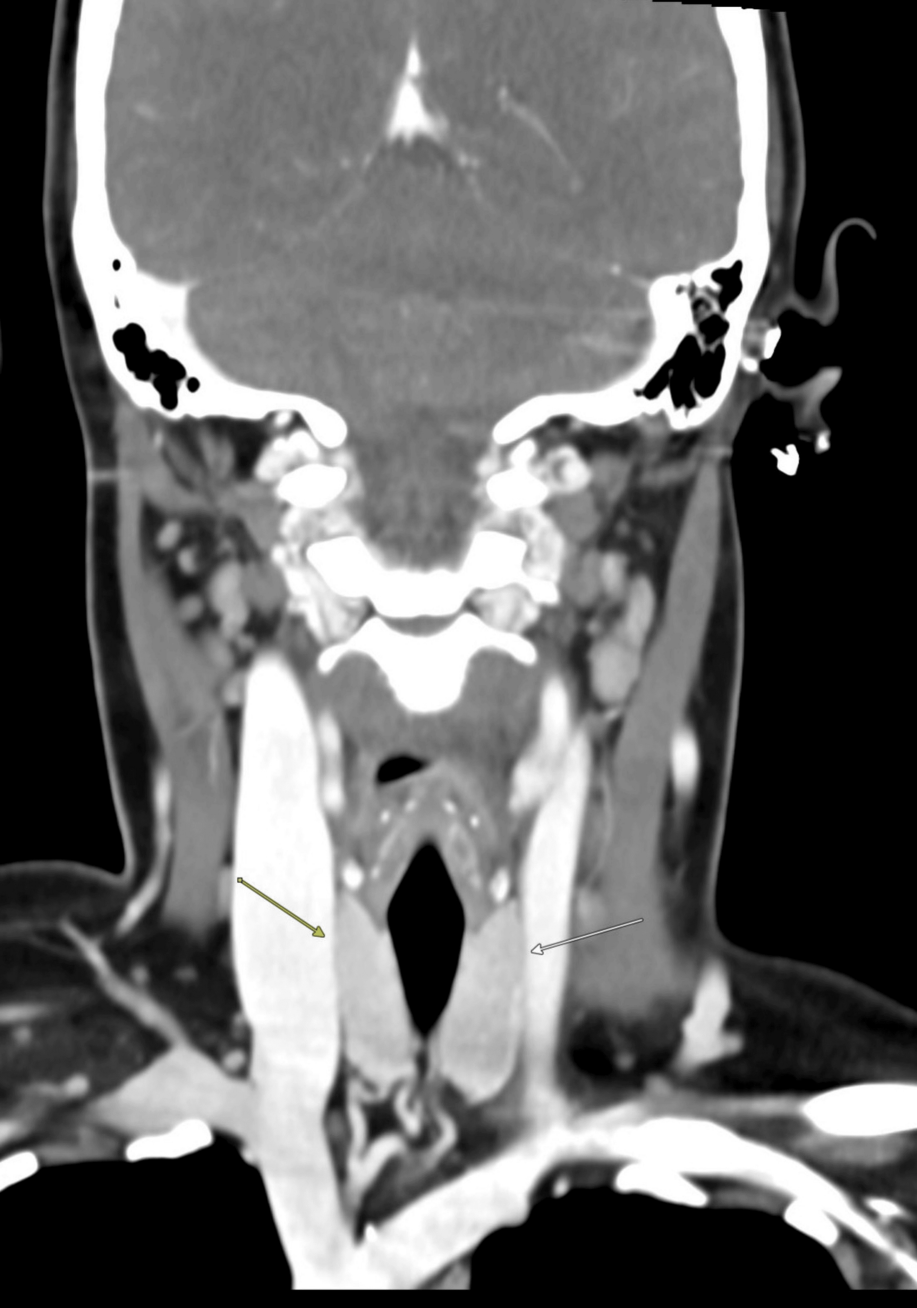
Coronal computed tomography image demonstrating bilaterally homogeneous thyroid lobes (arrows)

Computed tomography of the soft tissues of the neck demonstrated bilateral palatine tonsillar enlargement consistent with acute tonsillitis, with left-sided phlegmonous changes and no drainable abscess (Figures 3, 4). The thyroid gland appeared homogeneous on imaging (Figures 5, 6). Reactive cervical lymphadenopathy was also noted. Evaluation for infectious etiologies included negative group A streptococcal testing, negative mononucleosis screening, and a throat culture that did not identify pathogenic organisms. Diagnostic viral testing (e.g., COVID-19, influenza, RSV, or cytomegalovirus PCR) was not performed. The patient was treated empirically with antibiotics and corticosteroids, with subsequent clinical improvement in pharyngeal symptoms. Granulocyte colony-stimulating factor (G-CSF) was not administered, as hematologic recovery began rapidly.

A repeat complete blood count obtained approximately 24 hours later demonstrated early improvement in neutrophil counts, although neutropenia persisted. Concurrent thyroid testing showed biochemical worsening of hyperthyroidism following methimazole withdrawal. Longitudinal trends in hematologic parameters, thyroid function tests, and methimazole dosing are summarized in Table 1.

**Table 1:** Hematologic and thyroid function trends during methimazole therapy Reference ranges varied by laboratory and are reported as provided at the time of testing: white blood cell count reference range, 3.8-11.0 × 10³/µL; absolute neutrophil count, 1.5-8.8 × 10³/µL; thyroid-stimulating hormone, 0.27-4.5 µIU/mL; free thyroxine, 0.70-1.68 ng/dL; an absolute neutrophil count below 0.5 × 10³/µL meets laboratory criteria for agranulocytosis. “Improving” indicates a documented trend toward normalization relative to the prior nadir or peak. “Worsening” indicates biochemical deterioration relative to prior values.

Timepoint	White blood cell count (×10³/µL)	Absolute neutrophil count (×10³/µL)	Thyroid-Stimulating Hormone (µIU/mL)	Free thyroxine (ng/dL)	Methimazole dose
Six months prior to presentation	4.4	1.5	0.262	0.95	Not receiving therapy
Initial presentation	Not available	Not available	<0.005	2.86	Initiated at 105 mg per week
Approximately one month	Not available	Not available	0.03	0.94	Continued
Emergency department presentation	1.5	0.06	Not available	Not available	Held
Approximately 24 hours later	1.8	0.14	0.018	1.42	Discontinued
Clinical decision point	Improving	Improving	Improving	Worsening	Restarted at 80 mg per week
Approximately two weeks	4.4	1.82	Not available	0.62	Reduced to 50 mg per week
Approximately six weeks	6.3	2.89	2.26	Not available	Reduced to 40 mg per week
Most recent follow-up	Not available	Not available	1.67	Not available	Continued

Although the ANC nadir met the criteria for agranulocytosis, the rapid hematologic improvement within approximately 24 hours, clear temporal association with an acute infectious illness, and absence of continued decline raised concern for infection-associated transient neutropenia rather than classic immune-mediated methimazole-induced agranulocytosis. This distinction was clinically important, as continued interruption of antithyroid therapy was associated with worsening thyrotoxicosis.

Clinical management was further complicated by the patient’s self-pay status, which limited access to frequent in-person follow-up, intensive serial laboratory monitoring, and immediate definitive therapy. Propylthiouracil was not considered due to the risk of cross-reactivity in suspected antithyroid drug-associated neutropenia, consistent with current guideline recommendations. Given the rapid recovery of white blood cell counts, concurrent biochemical worsening of hyperthyroidism after methimazole discontinuation, and clinical features pointing toward an infection-associated etiology, a highly cautious trial of methimazole reintroduction was undertaken. This approach does not represent the standard of care in confirmed antithyroid drug-induced agranulocytosis. Rather, methimazole was reintroduced only because the clinical course was more consistent with infection-associated transient neutropenia and with explicit contingency planning for radioactive iodine therapy or thyroidectomy should hematologic or thyroid parameters fail to improve as expected.

Following extensive shared decision-making and objective evidence of early hematologic recovery, methimazole was cautiously reintroduced at a reduced total weekly dose of 80 mg, administered as 10 mg daily for six days per week and 20 mg on one day per week. This approach was undertaken under predefined stopping rules, with explicit plans for immediate and permanent discontinuation should the WBC or ANC worsen.

After methimazole reintroduction, complete blood counts and thyroid function tests were monitored closely at intervals determined by clinical judgment and laboratory trends. Two weeks later, laboratory testing demonstrated substantial hematologic recovery, with a WBC count of 4.4 × 10³/µL and an ANC of 1.82 × 10³/µL. FT4 had decreased to 0.62 ng/dL, indicating improving thyroid control with relative biochemical oversuppression, and the methimazole dose was reduced to 50 mg per week.

Approximately six weeks later, blood counts had normalized completely, with a WBC count of 6.3 × 10³/µL and an ANC of 2.89 × 10³/µL. Thyroid function continued to improve, with a TSH level of 2.26 µIU/mL, and methimazole was further reduced to 40 mg per week to maintain biochemical euthyroidism. At the most recent follow-up, TSH was 1.67 µIU/mL, and the patient remained clinically well, without recurrence of neutropenia or infectious complications.

The diagnosis of infection-associated transient neutropenia was therefore made clinically, as a diagnosis of exclusion, supported by objective evidence of acute tonsillitis documented on computed tomography, rapid spontaneous hematologic recovery, an appropriate infectious context, and sustained neutrophil normalization despite cautious methimazole continuation. The absence of confirmatory viral testing represents a limitation of this case.

## Discussion

Antithyroid drugs remain a cornerstone of therapy for Graves’ disease, with methimazole favored because of its efficacy, longer duration of action, and more favorable safety profile compared with propylthiouracil [1-3]. Among recognized adverse effects, agranulocytosis is the most feared complication, given its potential for rapid clinical deterioration and mortality. Accordingly, clinical guidelines uniformly recommend immediate and permanent discontinuation of methimazole once true antithyroid drug-induced agranulocytosis is confirmed, with avoidance of propylthiouracil because of the risk of cross-reactivity [1,2].

In contrast, infection-associated transient neutropenia represents a distinct and often reversible phenomenon that may closely mimic antithyroid drug-induced agranulocytosis on initial laboratory evaluation. Acute infectious or inflammatory stress can result in abrupt reductions in circulating neutrophil counts through multiple mechanisms, including immune-mediated peripheral destruction, transient bone marrow suppression, and neutrophil margination. During acute viral or inflammatory states, neutrophils may rapidly redistribute from the circulating pool to the marginal pool lining the vascular endothelium, leading to a markedly reduced absolute neutrophil count on peripheral blood sampling. Importantly, this redistribution can normalize quickly once the inciting stressor resolves, without requiring new marrow production.

Adding further diagnostic complexity, Graves’ disease itself has been associated with mild baseline leukopenia or neutropenia, even prior to initiation of antithyroid drug therapy. This intrinsic hematologic variability may lower the threshold at which patients develop clinically significant leukopenia during periods of physiologic stress or intercurrent illness, increasing the risk of misattributing transient neutropenia to drug toxicity.

The present case underscores the importance of hematologic trajectory, rather than absolute nadir alone, in determining etiology. Although the patient’s ANC met laboratory criteria for agranulocytosis, neutrophil recovery began within approximately 24 hours. Such rapid improvement is inconsistent with immune-mediated antithyroid drug-induced marrow failure, in which neutrophil recovery typically requires one to two weeks following drug discontinuation. The close temporal association with acute tonsillitis, absence of progressive decline, and sustained normalization of counts strongly supported an infection-associated process rather than classic methimazole toxicity.

Key clinical and laboratory features that help distinguish classic antithyroid drug-induced agranulocytosis from infection-associated transient neutropenia, including timing relative to drug exposure, trajectory of neutrophil recovery, infectious context, and response to continued therapy, are summarized in Table 2. This comparison highlights how reliance on a single laboratory value, without consideration of clinical context and recovery kinetics, may lead to premature diagnostic conclusions. Table 2 is synthesized from published literature on antithyroid drug-induced agranulocytosis and infection-associated transient neutropenia [1-7].

**Table 2:** Distinguishing features of classic antithyroid drug-induced agranulocytosis versus infection-associated transient neutropenia ATD: antithyroid drug.

Feature	Classic ATD-induced agranulocytosis	Infection-associated transient neutropenia
Timing relative to antithyroid drug initiation	Most commonly occurs within the first two to three months of therapy, although delayed cases have been reported	May occur at any time and often coincides with or follows an acute infectious illness
Depth of neutropenia	Frequently profound, with absolute neutrophil counts below 500/µL	Variable in severity; neutropenia may be marked but typically improves as the infection resolves
Laboratory trajectory without definitive intervention	Does not reliably improve without discontinuation of the offending drug; recurrence has been reported with re-exposure	Often improves rapidly, sometimes within days, as the underlying infection or inflammatory trigger resolves
Response to re-exposure or continuation of antithyroid therapy	Re-exposure is generally contraindicated, as relapse of agranulocytosis has been reported after drug restart	If neutropenia is not drug-mediated, neutrophil counts may remain stable after infection resolution, requiring close monitoring
Management implications	Immediate and permanent cessation of the antithyroid drug is recommended; propylthiouracil should be avoided because of cross-reactivity risk, and definitive therapy should be considered	Treat the underlying infection, closely monitor complete blood counts, and reassess causality to avoid unnecessary loss of effective antithyroid therapy

Management decisions in this case required careful balancing of competing risks. Shortly after methimazole discontinuation, biochemical hyperthyroidism worsened, illustrating the clinical consequences of prolonged interruption of antithyroid therapy. At the same time, guideline-directed management appropriately discourages rechallenge in confirmed agranulocytosis. Importantly, this case does not challenge those recommendations. Rather, the decision to cautiously reintroduce methimazole was made only after objective evidence of hematologic recovery, careful reassessment of causality, and extensive shared decision-making, and was undertaken with predefined stopping rules and close monitoring.

Propylthiouracil was not considered because of the risk of cross-reactivity in suspected antithyroid drug-associated neutropenia. Definitive therapies, including radioactive iodine ablation or thyroidectomy, were discussed as contingency options but were deferred because of active infection, biochemical worsening of hyperthyroidism following methimazole withdrawal, and patient-specific logistical and financial constraints. These real-world considerations highlight the complexity of managing Graves’ disease in settings where ideal monitoring and immediate definitive therapy may not be feasible.

Following methimazole reintroduction, complete blood counts and thyroid function tests were monitored closely based on clinical judgment and laboratory stability. Neutrophil counts normalized and remained stable, and thyroid indices improved with dose titration, further supporting the interpretation of transient infection-associated neutropenia rather than persistent drug toxicity.

This report has important limitations. Diagnostic viral testing was not performed, and the diagnosis of infection-associated transient neutropenia therefore remains a clinical diagnosis of exclusion, supported by objective evidence of acute tonsillitis documented on computed tomography and the rapid hematologic recovery observed. Additionally, as a single-patient case report, these observations cannot be generalized and should not be extrapolated as a management strategy. Rather, the findings emphasize the importance of disciplined clinical judgment, careful interpretation of laboratory trends, and strict adherence to established guidelines when faced with diagnostic uncertainty.

In summary, this case illustrates that severe neutropenia occurring during methimazole therapy does not invariably represent permanent antithyroid drug toxicity. Rapid hematologic recovery, infectious context, underlying Graves’-related hematologic variability, and trajectory of laboratory values are critical in distinguishing transient neutropenia from true agranulocytosis. While permanent discontinuation remains the standard of care in confirmed cases, thoughtful, patient-centered decision-making grounded in objective trends may be required when real-world constraints complicate management.

## Conclusions

Severe neutropenia occurring during methimazole therapy does not invariably represent permanent antithyroid drug-induced agranulocytosis. This case illustrates that careful interpretation of hematologic trajectory, clinical context, and timing of recovery is essential in distinguishing transient infection-associated neutropenia from true methimazole toxicity. Rapid neutrophil recovery, particularly within 24 hours, strongly argues against immune-mediated marrow failure and should prompt reassessment of causality.

Importantly, this report does not advocate methimazole rechallenge in confirmed agranulocytosis. Permanent discontinuation of antithyroid drug therapy remains the standard of care in such cases. In this patient, methimazole reintroduction was undertaken only after objective evidence of hematologic recovery, a clinical course more consistent with infection-associated transient neutropenia, and extensive shared decision-making under predefined stopping rules in a constrained real-world setting. As a single-patient case report, these observations are not generalizable and should not be extrapolated as a management strategy. Rather, the findings emphasize the importance of disciplined clinical judgment, careful trend-based interpretation of laboratory data, and strict adherence to established guidelines when managing diagnostic uncertainty, particularly when ideal monitoring or definitive therapies are not immediately feasible.
